# Gerald Grace

**Published:** 1914-09

**Authors:** 


### GERALD GRACE, M.R.C.S., L.R.C.P.

THE death of Mr. Gerald Grace took place in South Africa as the result of a singularly tragic incident on Sept. 17th. For some time the Witwatersrand had been terrorised by a band of desperadoes. Mr. Grace was travelling on the Witwatersrand reef in a motor-car resembling that in which the gang was believed to be escaping. The police challenged the car, but, according to the account in the *Rand Daily Mail*, Johannesburg, "a strong wind was blowing at the time; in fact, a howling dust storm had just spent itself. Mr. Grace, it seems, could not have understood the signal, for, according to the statement of a civilian, who, with three others, was a witness of the occurrence, the doctor appeared to lower his head, and the car shot forward. The mounted police then fired three shots in quick succession, and the car was seen to swerve on to the side of the road. Apparently after the first shot rang out the doctor, realising the situation and his peril at the same time, slowed down and stopped, but one of the shots did its deadly work."

The court of inquiry has exonerated the police from any blame for the sad occurrence.

Gerald Grace was 47 years of age, and received his early education at the Bristol Cathedral School. He subsequently studied medicine at the Royal Infirmary and Guy's Hospital. As a medical student, he displayed uncommon talents as a conjurer and entertainer. The shrewd exercise of his gifts in this direction enabled him, whilst studying medicine, to earn enough by his entertainments to defray the expenses of his medical education. After qualifying he became ship surgeon on the Union Castle Line, where his versatility won him wide popularity and many friends. On the outbreak of the South African War he served as an army surgeon. At the end of the war he married and settled in practice, with no small success at Springs, of which town he was mayor at the time of his death. As a medical student he was a great favourite amongst his contemporaries and all who knew him. He was a man of uncommon parts, possessed of unbounded humour, and made rare good company. He will be greatly missed, and we feel the deepest sympathy with his family at the tragedy which has cut short his career. We regret to hear that Mrs. Grace, to whom he had only been married twelve months, herself received a bullet wound.

---

### Local Medical Notes.

